# Immunotherapy: A Potential Approach for High-Grade Spinal Cord Astrocytomas

**DOI:** 10.3389/fimmu.2020.582828

**Published:** 2021-02-18

**Authors:** Jie Hu, Tie Liu, Bo Han, Shishan Tan, Hua Guo, Yu Xin

**Affiliations:** ^1^ Department of Neurosurgery, Beijing Tiantan Hospital, Capital Medical University, Beijing, China; ^2^ Department of Neurosurgery, The Second Affiliated Hospital of Nanchang University, Nanchang, China

**Keywords:** spinal cord astrocytomas, immunotherapy, immune checkpoint inhibitors, CAR-T therapy, therapeutic vaccines therapy, K27M-mutant histone H3

## Abstract

Spinal cord astrocytomas (SCAs) account for 6–8% of all primary spinal cord tumors. For high-grade SCAs, the prognosis is often poor with conventional therapy, thus the urgent need for novel treatments to improve patient survival. Immunotherapy is a promising therapeutic strategy and has been used to treat cancer in recent years. Several clinical trials have evaluated immunotherapy for intracranial gliomas, providing evidence for immunotherapy-mediated ability to inhibit tumor growth. Given the unique microenvironment and molecular biology of the spinal cord, this review will offer new perspectives on moving toward the application of successful immunotherapy for SCAs based on the latest studies and literature. Furthermore, we will discuss the challenges associated with immunotherapy in SCAs, propose prospects for future research, and provide a periodic summary of the current state of immunotherapy for SCAs immunotherapy.

## Introduction and Current Management of Spinal Cord Astrocytomas

Spinal cord astrocytomas (SCAs) comprise approximately 6–8% of primary spinal cord tumors (1) and can be divided into the following categories based on the 2016 World Health Organization (WHO) classification: pilocytic astrocytomas (PAs, Grade I), diffuse astrocytomas (DAs, Grade II), anaplastic astrocytomas (AAs, Grade III), and glioblastomas multiforme (GBMs, Grade IV) (2, 3). In addition, a new histological diagnosis has been proposed to include diffuse midline gliomas (DMGs), with H3K27M mutant, which are found in the spinal cord, brainstem, pineal region, and thalamus (2). Generally, 75% of primary SCAs are low-grade (WHO grade I–II), while the remaining 25% are high-grade (WHO grade III–IV) tumors (4).

The current standard-of-care therapy for SCAs involves maximal safe surgical resection, followed by radiotherapy and chemotherapy. For low-grade primary SCAs, gross total resection is considered the first treatment choice and has an excellent local control rate (5). However, the value of aggressive resection in high-grade SCAs is unknown and cannot be recommended since the infiltrative nature of these SCAs frequently limits the extent of resection. For these tumors, aggressive surgical removal is not associated with any significant survival benefit, with a mortality rate of up to 70% at 6 months (6).

In conclusion, SCAs are rare diseases and are challenging to treat. For high-grade SCAs, the currently available therapies seem to do little to improve the survival of patients. In the future, better therapeutic options are needed to treat high-grade SCAs to prolong the patient’s life, and immunotherapy might be a potential treatment for those patients.

## Spinal Cord Microenvironment and Its Impact on Tumor Biology

The spinal cord microenvironment not only plays an important role in the process of tumor occurrence, development, and metastasis but also influences therapeutic effects (7).

In the chapter “Spinal Cord Tumor Microenvironment” in TME in organs, Ellis and colleagues have demonstrated that the TME in the spinal cord and brain are different (8). In their study, the author found that the same source of tumor cells transplanted into the spinal cord tissue showed lower tumor growth than in the brain, which led the researchers to speculate that the occurrence and development of glioma may be due to the different environment present in the brain and spinal cord. In addition, the literature provides evidence that genetic changes in intramedullary astrocytomas are less frequent than in intracranial astrocytomas (8). One possible explanation may be the relatively small spinal canal volume, which makes the tumor more prone to symptoms at an earlier stage of development.

As we all know, the spinal cord is located in the spinal canal, composed of gray and white matter, covered from inside to outside by the pia mater, arachnoid mater, and dural mater. The subarachnoid space is filled with cerebrospinal fluid, which provides mechanical and immune protection for the spinal cord. The existence of the blood-spinal cord barrier (BSCB) makes the spinal cord form a relatively independent microenvironment, which strictly regulates metabolism and immune transport to the spinal cord parenchyma as the same as the function of the blood-brain barrier. Although the spinal cord, like the brain, has long been considered immune exclusion zone, this view has been challenged in recent years.

Glial cells are the most abundant cell types in the spinal cord, including astrocytes, oligodendrocytes, ependymal cells, and microglias. The glia-neuron ratio in the spinal cord is suspected to be much higher than in the brain (9). Among them, astrocytes are the most common cell type that play an important role in the normal functioning of the spinal cord and also participate in the occurrence and development of tumors in many ways. Although relevant studies have been carried out in intracranial gliomas, due to the large regional heterogeneity of astrocytes, intracranial studies cannot be fully applied to SCAs, and relevant studies need to be further carried out.

Lymphoid cells can provide long-term immune monitoring and play an important role in maintaining homeostasis and tumor development. In this context, the concept of checkpoint inhibitor was proposed and received more attention. The discovery of PD-1, PD-L1, CTLA-4, and other molecules and the development of related drugs have been effective in some tumors. In CNS malignancies, tumor molecules can avoid detection by recruiting and coordinating T lymphocytes, and transform the immune system from protective to toxic (10). In spinal cord, the expression rate of PD-L1 is about 20% according to one study (11). Clinical trials targeting immune checkpoints have been carried out in intracranial gliomas, although the results have not been satisfactory and the study of spinal astrocytomas is still in the blank.

In a word, compared to studies on the intracranial glioma microenvironment, there is still a lack of data specific to the spinal cord, which indicates that future research is necessary. With the development of relevant studies and the gradual understanding of the spinal cord microenvironment, the treatment of spinal cord tumor will also change accordingly, making treatment more targeted and efficient.

## Molecular Biology of Spinal Cord Astrocytomas

Characteristic molecular markers in tumor tissues are important for judging tumor pathological grade and treatment prognosis. Therefore, it is of great importance to search for characteristic molecular indicators of SCAs, especially high-grade. Below, we summarize specific biomarkers of SCAs (**Table 1**).

**Table 1:** Molecular Markers of Spinal Cord Astrocytomas (SCAs).

Gene/Mutation	Mutation frequency in SCAs				Comments
	PAs (Grade I)	DAs(Grade II)	AAs(Grade III)	GBMs(Grade IV)	
BRAF KIAA1549	32% (12)	Rare			More common in PAs in the spinal cord and the basilar parts of the brain (13).
BRAF V600E	48% (12)	Rare			Most frequently found in supratentorial PAs.
CDKN2A	+				Crucial to SCAs and, in particular, PAs (13).
H3K27M	–	52% (adults) (14) 54% (children) (14)			SCAs with K27M mutation have malignant biological behavior and are highly invasive (15). Indicated as the pathological basis for high-grade SCAs and is an important indicator of poor prognosis (16).
TERT promoter	–	22% (17)			TERT promoter mutations are associated with shorter survival in SCAs patients (17, 18).
TP53	–	60% (19)			More aggressive disease course (20). Revealed in 60%–67% of grade III-IV SCAs (19, 21).
PTEN	Extremely rare				Only sporadic reports are known in grade III–IV (22).

Green refers to the genes that are related to low-grade (Grade I–II) SCAs; Blue refers to genes that are related to high-grade (Grade III–IV) SCAs. The symbols “+” and “–” stand for the presence or absence of a mutation in SCAs.

## H3K27M

The K27M mutation is one of two mutually exclusive variants of the H3F3A gene. Gliomas harboring this mutation mainly localize to midline structures, such as the thalamus, brainstem, and spinal cord, and are prevalent in adolescents and children with malignant biological characteristics (16, 23). The H3K27M mutation lacks typical cellular genetic abnormalities and is usually found in high-grade gliomas characterized by unusually aggressive tumor progression (24), even if it not classified histologically as low-grade astrocytomas (15).

The K27M mutation in patients with SCAs is associated with grade III–IV tumors. Chai et al. (17) revealed the K27M mutation was detected in 42.1% of cases (n = 83) of SCAs. Nagaishi et al. (23) showed similar data. Thus, this mutation is often associated with grade III–IV SCAs. Another study showed approximately the same mutation frequency rate for grade III–IV astrocytomas in adults and children (52% and 54%; n = 11 and 19, respectively) (14). It should be noted that K27M is absent in other types of malignant tumors, so it may be a pathological feature of primary malignant SCAs and may also serve as an indicator of the worst prognosis (16). Research on H3K27M as an important therapeutic target is under way, which will be described below.

## Immunotherapy: A Potential Approach for High-Grade SCAs

In recent years, alongside the advancement of research and the continuous improvements in technology, tumor immunotherapy has gradually become an important treatment modality in addition to surgical treatment and postoperative radiotherapy and chemotherapy. Immunotherapy has played an increasingly important role in the treatment of hematological malignancies and melanoma and other malignancies. Immunotherapeutic agents have been approved for marketing and have benefited a number of patients. Immunotherapy is also at the experimental stage in glioma. Recent novel advances in immunotherapy include immune checkpoint inhibitors, chimeric antigen receptor (CAR)-T therapy, and vaccine therapy (25). The application of immunotherapy to the treatment of high-grade spinal cord astrocytomas is promising, and the results may be exciting, although at present, there are still considerable challenges. Below, we will summarize the current status of immunotherapy in SCAs and the problems to be resolved in the future.

## Immune Checkpoint Inhibitors

Immunocheckpoint inhibitors have played an important role in the treatment of many kinds of tumors. At present, common immune checkpoints include PD-1、PD-L1、CTLA-4. These molecules have been shown to be highly expressed on the surface of intracranial glioma molecules, and their expression level is positively correlated with tumor grade (26). In preclinical studies, relevant studies have shown that anti-PD-1, anti-PD-L1, and anti-CTLA-4 can achieve tumor survival in 50%、20%, and 15% of mice respectively when treated with tumor model alone. Combined use of anti-PD-1 and anti-CTLA-4 can effectively prevent tumor growth (27). However, due to the differences between the animal model and the actual tumor microenvironment, the data of relevant clinical studies were unsatisfactory, and most of them focused on stage I and II. Nivolumab, a monoclonal antibody used to block PD-1, is currently undergoing clinical trials and has shown that its use alone does not extend overall survival (28). In another trial, pembrolizumab, another anti-PD-1, has been used to assess immune response and survival analysis in patients with GBM after surgical resection as a neoadjuvant therapy. The results of this study suggest that the overall survival of patients can be prolonged (29). In general, the effect of immunocheckpoint inhibitors as a single treatment is limited, but combined with other treatment methods, such as targeted drugs, radiotherapy, chemotherapy, and so on, the therapeutic effect may be improved. Although this approach is currently rarely used in high-grade spinal cord astrocytomas, relevant treatment strategies can be used for reference.

## CAR-T Cell Therapy

Chimeric antigen receptor (CAR) T cell therapy is an example of adoptive cell therapy and is a promising immunotherapy approach. CARs are synthetic receptors that alter the specificity, function, and metabolism of T cells (30). A major advantage of CAR-T therapy is that through the short-chain variable fragment (ScFv), T cells can directly recognize tumor surface specific antigens without the need for major histocompatibility complex (MHC) complex antigens, effectively overcoming the limitations of previous TCR-T cells (31).

CAR-T therapy was first described in the mid-1980s (32). However, only in recent years has it been successfully introduced into clinical practice. In 2007, the FDA approved the first application of an anti-CD19-CAR-T clinical trial. The results were encouraging (33), and thus scientists were stimulated to apply this approach to central nervous system tumors. In adult glioblastomas, CAR-T have targeted specific antigens such as human epidermal growth factor receptor 2 (HER2) (34), epidermal growth factor receptor (EGFR) vIII (35, 36), interleukin (IL)-13Rα2 (37), and ephrin type-A receptor 2 (EphA2) (38), and preliminary results are encouraging. Although clinical conversion of these agents is not yet possible, the results have provided scientists with considerable confidence regarding their promising clinical implication.

However, the specific targets identified in glioblastomas of the brain are not suitable for high-grade SCAs. Encouragingly, a recent study (39) has suggested that H3K27M mutation may be a potential target for immunotherapy of high-grade SCAs. The study (39) was conducted in the broad framework of diffuse midline gliomas. The disialoganglioside GD2, a group of galactose-containing complex-lipid structures found on membranes of cells, was identified and confirmed as being specific and highly expressed in the pathological tissues of patient derived diffuse midline glioma, but not on the surface of normal cells. In the H3K27M+DMG orthotopic xenograft model derived from five independent patients (including spinal cord sources), GD2-targeted CAR-T cells could produce the cytokines inteferon(IFN)-γ and interleukin-2 (IL-2) and selectively killed tumor cells. The transplanted tumor cells were significantly reduced in size, which was obviously encouraging. However, the neurotoxicity of CAR-T cell therapy is also of concern. GD2-CAR-T cells in mice showed significant toxicity over the maximum therapeutic period. The death of mice was attributed to local third ventricle compression caused by inflammatory infiltration, rather than the targeted, non-tumor toxicity of specific GD2-CAR-T cells. The author concluded that due to the particular anatomical site of the midline structure, the swelling caused by neuroinflammation is often not tolerated (39); thus, more detailed clinical testing and intensive neuroprotective treatment are needed. Even so, the risk of cerebral hernia may not be effectively reduced. Although the above studies in the mouse xenograft model suggest that GD2-specific CAR T cell therapy has promising therapeutic potential and that most mice can tolerate the inflammatory response associated with the activity of CAR T cells, whether this model is able to predict human outcomes remains to be determined.

## Therapeutic Vaccines Therapy

Therapeutic vaccines are also a promising therapeutic approach, but their exact efficacy is unclear (40). Therapeutic vaccines therapy can be roughly divided into several categories: peptide vaccines, dendritic cell vaccines, tumor cell vaccines, and neoantigen vaccines (41). Importantly, the use of adjuvants is crucial in immunologically privileged environments such as the brain and spinal cord, because the lack of resident immune cells in the CNS may hamper an effective immune response. As a result, vaccines are being developed and refined to adapt to the immunosuppressive tumor microenvironment (TME).

H3K27M is a specific biological marker for primary high-level SCAs and a promising target for immunotherapy. H3K27M-targeted vaccines have shown good therapeutic effects in preclinical models (42, 43). Ochs et al. (42) have done a lot of work in this regard, and their data have provided the basis for the further development of a vaccine against H3K27M. Their results mainly confirmed that H3K27M can be targeted by mutant specific peptide vaccines and can induce specific T cell responses. Besides, the H3K27M peptide vaccine significantly reduced tumor growth in the transplanted tumor mouse model, and induced effective CTL and Th-1 anti-tumor immune responses. The authors’ findings provided a solid theoretical basis for vaccine development targeting the H3K27M mutation. It should also be noted, however, that the H3K27M homogenic hypodermic sarcoma model was used in their studies, and that an MHC humanized *in situ* glioma model was lacking to demonstrate the efficacy and reliability of the vaccine.

Limited by the few specific epitopes available, there have been few studies on targeted vaccines for high-grade SCAs. Thus, current treatment of targeted vaccines for SCAs is still in its infancy stage. H3K27M has been shown to be an effective immunogenic epitope for SCAs and represents a promising breakthrough point for the development of targeted vaccines for SCAs in the future. It is also hoped that new immunogenic epitopes will be discovered and confirmed in the future.

## Discussion

SCAs, especially high-grade SCAs, tend to occur in adolescents and have a high degree of malignancy and poor prognosis. The existing treatment methods are of little help to improve patient survival. Immunotherapy has shown great anti-tumor potential in other malignant tumors, and it has potential therapeutic significance for spinal cord patients based on the data available. Even so, the current research is limited and mainly focuses on experimental studies in animal models, which is mainly due to the following (44): the incidence of high-grade spinal cord astrocytomas is very low compared with that of intracranial gliomas; thus, it is difficult to obtain enough samples for a full and objective analysis. Furthermore, the spinal cord controls the upper and lower limbs, and because high-grade gliomas are often characterized by invasive growth that is not clearly distinguishable from normal tissue structure, it is difficult to obtain enough tissue for detailed analysis. Therefore, regional or international cooperation is desirable. Since spinal cord astrocytomas are diseases with a low incidence, studies at this stage are more focused on single centers, with a limited number of cases, and the conclusions drawn are often incomplete. Therefore, in the future, it is expected to enrich the pathological sample size of spinal cord astrocytomas through the cooperation of all parties, so as to conduct a systematic and comprehensive analysis and continuously deepen the understanding of this disease. Only with a deeper understanding of the disease can we better diagnose and treat it.

There are still many difficulties and challenges that need to be addressed in the future, including (i) antigen escape and paucity of tumor specific antigens; (ii) drug delivery crossing the blood spinal cord barrier (BSCB); (iii) neurotoxicity on the spinal cord; and (iv) the unique immune cohort in SCAs.

The number of specific antigens identified in SCAs, especially high-grade SCAs, is too small at present, which seriously restricts the development of relevant immunotherapy drugs in SCAs. Recent studies have shown that the genomic landscape of SCAs is significantly different from that of intracranial astrocytomas, with unique and highly recurrent mutations in the genes encoding the H3 variant of the histone (45, 46). Other genes identified contain TP53 and the TERT promoter. With this knowledge, a new approach for the discovery of tumor-specific targets for SCAs is necessary, instead of treating and testing intracranial astrocytomas as in the past. Scientists will need to determine how to identify new specific molecular markers expressed in SCAs tissue samples that can more effectively guide the immune system toward cancer eradication.

Antigenic escape is a thorny issue for scientists and clinic doctors. Antigen escape has been identified as an important cause of drug resistance and tumor relapse in acquired leukemia (33). This problem may also be encountered in the immunotherapy of SCAs. In view of the limited number of targeted antigens available at present, only single antigens can be used for CAR-T preparation, which further increases the probability of antigen escape and reduces the anti-tumor effectiveness of drugs, while the existence of tumor heterogeneity makes this problem more prominent. It is no exaggeration to say that the task of finding more specific targets is urgent so as to include effective and safe immunotherapy.

How to improve the targeted drug’s ability to cross the BSCB, so as to better reach the lesion to improve efficacy, is a question that future research should consider. Methods to improve the penetration of drugs through the blood-brain barrier (BBB) can be used for reference. A potential approach would the use of nanoparticle systems that can co-opt existing signaling pathways (47). Physically breaching the BBB may be another approach, for example, by reversibly doing so using pulsed ultrasonic sound waves; this is already an option clinically available for glioma treatment (48). The BSCB exerts roughly similar functions as BBB, but there is evidence that the interface between the blood circulation and CNS is not evenly distributed along the entire neural axis (49, 50); thus, whether these methods can be directly applied to BSCB, or modified according to the specific characteristics of the BSCB, using biological engineering technology so as to improve the bioavailability of effective drugs to penetrate BSCB required investigation.

It remains to be seen whether the drugs used for immunotherapy cause potential damage to the CNS. Unlike other organs, the CNS cannot tolerate even minimal autoimmune responses (51). There is a lack of data and analysis on the long-term effects of immunotherapy, and the results remain unknown. In the current model, no significant CNS adverse events have been reported for CAR and therapeutic vaccine therapy. However, the potential damage to patients from immunotherapy is inestimable due to differences between the mouse model and humans. At the same time, given that patients with primary CNS tumors may have a different disease burden than patients with leukemia secondary CNS diseases, the risk of potential neurotoxicity in the SCA population may be higher (25). The question of how to monitor the response of SCAs also needs to be addressed.

Lastly, how to obtain enough peptides to antigen-presenting cells (APCs) and the subsequent immune response cascade will be a major challenge for peptide vaccines in the future (52). The CNS had been considered an immunologically privileged site for a long time (52). However, according to existing studies, a series of unique immune characteristics exist in CNS, including the natural expression of immunosuppressant factors such as transforming growth factor (TGF) and IL-10, low expression of MHCs, lack of effective APCs, and the presence of the BBB and the BSCB. Although T cells and antibodies can be exposed to CNS antigens in gliomas, the lack of adequate APCs in the spinal TME may affect the effectiveness of some immunotherapies, including vaccines (52). Increasing APCs recruitment at injection sites has been explored through a variety of adjuvants; in addition, designing continuous APCs recruitment before and during vaccination may have good therapeutic potential.

The application of immunotherapy to SCAs presents many unique challenges, in particular how to monitor responses and the effects of treatment on the spinal cord. We hope that future development in immunotherapy will allow improved anti-tumor efficacy of highly malignant SCAs. As a result, the question of how to combine this new treatment with traditional therapies will become increasingly important.

## Conclusion

High-grade SCAs is an aggressive tumor with malignant biological behavior; it is more common in adolescents and children and has a very poor prognosis. Due to its lower incidence than brain glioma and invasive growth, the survival rates of these patients have not been significantly improved even with currently available standard treatment. Immunotherapy is considered as a promising approach due to the cytotoxic potential of the immune system and the precision of molecular guidance (53). However, while immunotherapy has shown promising results in other cancers, little progress has been made in tumors of the CNS, including brain gliomas and SCAs. In the future, immunotherapy of SCAs will need to take into consideration the penetration of the BSCB, the escape mechanism of immune antigens, the lack of known specific targets, the neurotoxicity of the drug to the normal spinal cord structures, and the ability to enhance the local immune response. Researchers need to combine more advanced technology with multi-center collaborations to further expand the sample size to better understand the microenvironmental and biological characteristics of high-grade SCAs, and to use this information to develop combinations of multiple immunotherapies as a meaningful therapy, able to overcome poor clinical results in this subgroup (25).

## Author Contributions

JH and TL: manuscript preparation and wrote the main part of the manuscript. ST: wrote parts of the manuscript and manuscript editing. BH and GH: helped to perform the analysis with constructive discussions. YX: critically reviewed the manuscript and gave many professional suggestions. All authors contributed to the article and approved the submitted version.

## Conflict of Interest

The authors declare that the research was conducted in the absence of any commercial or financial relationships that could be construed as a potential conflict of interest.
